# Supplementary material for: Effectiveness of COVID-19 Vaccines in People with Severe Mental Illness: A Systematic Review and Meta-Analysis
Source: Vaccines (Basel). 2024 Sep 18;12(9):1064. doi: 10.3390/vaccines12091064 (PMC11436207; doi:10.3390/vaccines12091064)
Supplement: Supplementary file 1 [file vaccines-12-01064-s001.zip › vaccines-3145217-supplementary.pdf]

Supplementary Materials

METHOD S1 SEARCH TERMS 1

TABLE S1 BASIC CHARACTERISTICS OF INCLUDED STUDIES 3

TABLE S2 QUALITY ASSESSMENT OF INCLUDED STUDIES (SCORES FROM NEWCASTLE–OTTAWA SCALE) 9

FIGURE S1 LEAVE-ONE-OUT FOREST PLOT ILLUSTRATING ODDS RATIO OF COVID-19-RELATED OUTCOMES IN PATIENTS WITH SEVERE MENTAL ILLNESS 10

FIGURE S2 FUNNEL PLOT FOR PUBLICATION BIAS FOR POOLED COVID-19-RELATED OUTCOMES POST VACCINATION IN PATIENTS WITH SEVERE MENTAL 11

TABLE S3 RESULTS OF EGGER TEST FOR COVID-19-RELATED OUTCOMES 12

REFERENCE 13

Method S1 Search Terms

Search terms were derived from previously published systematic reviews and meta-analyses related to the COVID-19 vaccine or SMI.

The search terms of PubMed, as an example, were listed as follows:

("COVID-19"[Title/Abstract] OR "COVID19"[Title/Abstract] OR "COVID2019"[Title/Abstract] OR "covid 2019"[Title/Abstract] OR "novel coronavirus"[Title/Abstract] OR "new coronavirus"[Title/Abstract] OR "novel corona virus"[Title/Abstract] OR "new corona virus"[Title/Abstract] OR "SARS-CoV-2"[Title/Abstract] OR "SARSCoV2"[Title/Abstract] OR "SARS-CoV2"[Title/Abstract] OR "2019nCoV"[Title/Abstract] OR "2019-nCoV"[Title/Abstract] OR "2019 coronavirus"[Title/Abstract] OR "2019 corona virus"[Title/Abstract] OR "coronavirus disease 2019"[Title/Abstract] OR "severe acute respiratory syndrome coronavirus 2"[Title/Abstract] OR "sars-coronavirus-2"[Title/Abstract] OR "corona virus disease 2019"[Title/Abstract]) AND ("covid 19 vaccines"[MeSH Terms] OR "covid 19 vaccine"[Title/Abstract] OR "mrna 1273 vaccine"[Title/Abstract] OR "mrna vaccine"[Title/Abstract] OR "mrna covid 19 vaccines"[Title/Abstract] OR "chadox1 covid 19 vaccine"[Title/Abstract] OR "ad5 ncov vaccine"[Title/Abstract] OR "Ad5-nCoV"[Title/Abstract] OR "covid 19 aapc vaccine"[Supplementary Concept] OR "ad26 cov2 s vaccine"[Title/Abstract] OR "adenoviral vector vaccine"[Title/Abstract] OR "bnt162 vaccine"[Supplementary Concept] OR "BNT162b2"[Title/Abstract] OR "BNT162"[Title/Abstract] OR "CoronaVac"[Title/Abstract] OR "vaccin*"[Title/Abstract]) AND ("severe mental illness"[Title/Abstract] OR "serious mental illness"[Title/Abstract] OR "psychiatric disease"[Title/Abstract] OR "schizophrenia*"[Title/Abstract] OR "schizophrenic disorder*"[Title/Abstract] OR "schizoaffective disorder*"[Title/Abstract] OR "psychosis"[Title/Abstract] OR "bipolar disorder*"[Title/Abstract] OR "bipolar affective disorder"[Title/Abstract] OR "manic depress*"[Title/Abstract] OR "major depress*"[Title/Abstract] OR "clinical depress*"[Title/Abstract] OR "depressive disorder"[Title/Abstract]) AND ((("clinical trial, phase iv"[Publication Type] OR "controlled clinical trial"[Publication Type] OR "randomized controlled trial"[Publication Type] OR "case control studies"[MeSH Terms] OR "retrospective studies"[MeSH Terms] OR "Retrospective"[Title/Abstract] OR "cohort studies"[MeSH Terms] OR "prospective studies"[MeSH Terms] OR "Prospective"[Title/Abstract] OR "longitudinal studies"[MeSH Terms] OR "follow up studies"[MeSH Terms] OR "follow up studies"[Title/Abstract] OR "cohort"[Title/Abstract] OR "test negative"[Title/Abstract] OR "observational cohort"[Title/Abstract] OR "test negative design"[Title/Abstract] OR "RCT"[Title/Abstract] OR "Randomized"[Title/Abstract] OR "randomised"[Title/Abstract] OR "randomly allocated"[Title/Abstract] OR "case-control"[Title/Abstract] OR "real world effectiveness"[Title/Abstract] OR "effectiveness"[Title/Abstract] OR

"impact"[Title/Abstract] OR "vaccine impact"[Title/Abstract]) NOT ("clinical trial, phase i"[Publication Type] OR "clinical trial, phase ii"[Publication Type])) NOT (("animals"[MeSH Terms:noexp] OR "animals"[All Fields]) NOT ("human s"[All Fields] OR "humans"[MeSH Terms] OR "humans"[All Fields])))

Table S1 Basic characteristics of included studies

First author, year	Country	Study design	Data source	Diagnosis codes of SMI	SMI group				Control group				Type of vaccines	Outcomes	Follow-up (Months)
					Subtype	Sample size	Mean age, mean (SD), y	Male, No. (%)	Topic	Sample size	Mean age, mean (SD), y	Male, No. (%)			
Tzur Bitan et al. [1], 2021	Israel	Cohort	Clalit Health Services	ICD-9 and ICD-10	SZ	4660	NR	NR	Controls	5316	NR	NR	NR	COVID-19 infection: SARS-CoV-2 infection;<br><br>COVID-19 hospitalization: defined as admission to a COVID-19 ward of a general hospital;<br><br>COVID-19 mortality: defined as registry of COVID-19 being the cause of death as determined by the Ministry of Health registry.	The total length of follow-up: 3
Semenzato et al. [2], 2022	France	Cohort	the national health insurance reimbursement database; the National Health Data System (SNDS)	ICD-10	Psychotics disorders (use of neuroleptics treatments)	381370	NR	NR	Non-comorbidity	13882319	NR	NR	mRNA vaccine or viral vector vaccines (One or two dose of mRNA BNT162b2 vaccine, mRNA-1273 vaccine, ChAdOx1 nCoV-19 vaccine)	COVID-19-related hospitalization: patients requiring admission specifically for SARS-CoV-2 infection.<br><br>COVID-19-related death: death during COVID-19-related hospitalization.	Average follow-up: 2.7

Table S1 Basic characteristics of included studies (continued)

First author, year	Country	Study design	Data source	the choice of diagnostic tool for SMI	SMI group				Control group				Type of vaccines	Outcomes	Follow-up (Months)
					Subtype	Sample size	Mean age, mean (SD), y	Male, No. (%)	Topic	Sample size	Mean age, mean (SD), y	Male, No. (%)			
Nishimi et al. [3], 2022	United States	Cohort	Administrative and electronic health records of US Department of Veterans Affairs	ICD-9-CM or ICD-10-CM	Psychotic disorder	7326	62 (13)	6628 (91)	Non-PD	128216	70 (12)	121214 (95)	mRNA vaccine or viral vector vaccines (Pfizer-BioNTech or Moderna or 1 dose of Johnson & Johnson–Janssen vaccine)	COVID-19 infection: defined as a positive SARS-CoV-2 test recorded in VA clinical notes (among individuals 14 days after their final SARS-CoV-2 vaccine dose).	The total length of follow-up: 11.5
					BD	9439	58 (14)	7442 (79)							
					MDD	84588	62 (14)	71713 (85)							
Piazza et al. [4], 2022	Italy	Cohort	National Vaccine Registry and regional administrative flows	ICD-9 CM: 295*,296.1*,296.3*,296.4*,296.5*,296.7,296.8*,297*,298.0,298.2,298.4,298.8,298.9,299.0*,299.1*,299.8*,299.9*	Psychosis	1088	median age: 54 (IQR 38-70)	500 (46.0)	Non-psychosis	222350	NR	102001 (45.9%)	mRNA vaccines(BNT162b2 and mRNA-1273 vaccines); viral vector vaccines (ChAdOx1 and Ad26.COV2.S); protein vaccines (NVX-CoV2373); and mixed vaccines	COVID-19 infection: tested positive for SARS-CoV-2.	The total length of follow-up: 8

Table S1 Basic characteristics of included studies (continued)

First author, year	Country	Study design	Data source	Diagnosis codes of SMI	SMI group				Control group				Type of vaccines	Outcomes	Follow-up (Months)
					Subtype	Sample size	Mean age, mean (SD), y	Male, No. (%)	Topic	Sample size	Mean age, mean (SD), y	Male, No. (%)			
Corrao et al. [5], 2022a	Italy	Case–control	The Regional Health Service	ICD-9 CM	Psychosis	983	NR	NR	Non-SMI	31130	NR	NR	mRNA vaccine or viral vector vaccines (two doses of Pfizer, Moderna or Oxford-AstraZeneca, or one dose of vaccine by Janssen)	COVID-19 infection: defined as nasopharynx-geal swab testing positive for SARS-CoV-2 via a PCR test.	Average follow up: 4.6
					BD	70	NR	NR							
					MDD	3809	NR	NR							
Corrao et al. [5], 2022a	Italy	Case–control	The Regional Health Service	ICD-9 CM	Psychosis	1424	NR	NR	Non-SMI	26919	NR	NR	mRNA vaccine or viral vector vaccines (two doses of Pfizer, Moderna or Oxford-AstraZeneca, or one dose of vaccine by Janssen)	COVID-19 hospital admission: COVID-19 hospital admission, including admission to an intensive care unit, or death.	Average follow up: 4.6
					MDD	4910	NR	NR							
Corrao et al. [6], 2022b	Italy	Case–control	The Regional Health Service	ICD-9 CM	Psychosis	1943	NR	NR	Non-SMI	41093	NR	NR	mRNA vaccine (third dose of mRNA vaccine manufactured by Pfizer or Moderna)	COVID-19 hospitalization: starting from at least 14 days after completing the scheduled vaccination (with three doses), experienced COVID-19 hospital admission, including those in an intensive care unit, or death.	Average follow up: 3.7
					BD	120	NR	NR							
					MDD	7436	NR	NR							

Table S1 Basic characteristics of included studies (continued)

First author, year	Country	Study design	Data source	Diagnosis codes of SMI	SMI group				Control group				Type of vaccines	Outcomes	Follow-up (Months)
					Subtype	Sample size	Mean age, mean (SD), y	Male, No. (%)	Topic	Sample size	Mean age, mean (SD), y	Male, No. (%)			
Zhu et al. [7], 2022	China	Cohort	7 provinces or municipalities of China	DSM-IV	SZ	813	NR	NR	HC	2034	NR	916(45)	Inactivated vaccine (BBIBP-CorV or Sinovac COVID-19 vaccine)	COVID-19 infection: defined by specific symptoms related to COVID-19, chest computed tomography scan demonstrating radiographic features of COVID-19, and positive nucleic acid testing for SARS-CoV-2.	The total length of follow-up: 1
					BD	581	NR	NR							
					MDD	640	NR	NR							
Montoya et al. [8], 2023	United States	Cohort	electronic health records from a data-sharing consortium including 984 nursing homes	ICD-10	Schizophrenia	1941	NR	NR	Non-schizophrenia	21231	NR	NR	mRNA vaccines (Pfizer–BioNTech or Moderna)	COVID-19 infection: ICD-10 codes U07.1 (COVID-19, virus identified), Z86.16 (history of COVID-19), and J12.82 (pneumonia due to COVID-19) documented in the EHR correlate highly with laboratory testing dates, and so these claims codes were used to identify COVID-19 infection.	Average follow up: 8.3
Ward et al. [9], 2023	United Kingdom	Cohort	Data from the 2021 Census linked to electronic health records	NR	SMI	1065440	NR	NR	Non-SMI	13586000	NR	NR	COVID-19 booster dose	COVID-19 mortality: defined as any International Classification of Diseases (ICD) death with codes U07.1 and U07.2.	The total length of follow-up: 7.4

Table S1 Basic characteristics of included studies (continued)

First author, year	Country	Study design	Data source	Diagnosis codes of SMI	SMI group				Control group				Type of vaccines	Outcomes	Follow-up (Months)
					Subtype	Sample size	Mean age, mean (SD), y	Male, No. (%)	Topic	Sample size	Mean age, mean (SD), y	Male, No. (%)			
Cheng et al. [10], 2023	China, Taiwan	Cohort	A single medical center in Taiwan	ICD-9: 295-298	psychiatric disorder	1148	51.3 (20.4)	415 (36.1%)	general patients	14635	59.2 (29.6)	7070 (48.3)	NR	COVID-19 hospitalization: Hospitalization to the medical center within 45 days following a positive PCR test. Severe illness was defined as the need for mechanical ventilation or intensive care unit admission. COVID-19 mortality: defined as a death record within 45 days following a positive PCR test for COVID-19.	Average follow up:1.5
Yiu et al. [11], 2023	China, Hong kong	Case–control	The Hospital Authority; The Department of Health; the Centre for Health Protection	ICD-9: 290.x–319.x	SZ	4758	NR	NR	Anxiety disorder (Non-SMI)	1529	NR	NR	mRNA vaccines or inactivated vaccine (1) one-dose BNT162b2, (2) one-dose CoronaVac, (3) two-doses BNT162b2, (4) two-doses CoronaVac, (5) three-doses BNT162b2, (6) three-doses CoronaVac, (7) two-doses BNT162b2 followed by one-dose CoronaVac (BBC), and (8) two-doses CoronaVac followed by one-dose BNT162b2 (CCB)	COVID-19 hospitalization was defined as hospitalization within 28 days of COVID-19 infection confirmed by polymerase chain reaction (PCR) tests.	Not available
					BD	436	NR	NR							
					MDD	7855	NR	NR							

Footnotes: SMI=severe mental illness; ICD-9-CM=International Classification of Diseases, Ninth Revision, Clinical Modification; ICD-10-CM=International Classification of Diseases, Tenth Revision, Clinical Modification; ICD-9=International Classification of Diseases, Ninth Revision; ICD-10=International Classification of Diseases, Tenth Revision; DSM-IV= Diagnostic and Statistical Manual of Mental Disorders, 4th ed; PD=psychiatric disorder; NR=not reported; SZ=schizophrenia; MDD=major depressive disorder; BD=bipolar disorder; HC=healthy control.

Table S2 Quality assessment of included studies (Scores from Newcastle–Ottawa Scale)

Author, year	Selection	Comparability	Exposure/Outcome	Total score
Tzur Bitan et al., 2021	****	**	**	8
Semenzato et al., 2022	****	**	***	9
Nishimi et al., 2022	***	**	***	8
Piazza et al., 2022	****	**	***	9
Corrao et al., 2022a	****	**	***	9
Corrao et al., 2022b	****	**	***	9
Zhu et al., 2022	***	**	**	7
Montoya et al., 2023	***	*	***	7
Ward et al., 2023	****	**	***	9
Cheng et al., 2023	****	*	***	8
Yiu et al., 2023	****	**	***	9

Footnotes: Quality assessment from three perspectives, including selection (4 stars), comparability (2 stars), and exposure/outcome (3 stars), with the highest quality of 9 stars.

Eight out of eleven studies rated high on the domain of selection, as the data of seven studies were derived from the electronic medical record based on national-level data, suggesting the representativeness of the population. In the domain of comparability, all studies received a score of at least 1, indicating the adequacy of controlling for potential confounders. Additionally, nine studies [2-6, 8-11] were evaluated as having the highest score in reporting outcomes, while the Tzur Bitan et al.[1] and Zhu et al.[7] studies were assessed as having with lower scores due to the insufficient length of follow-up for outcomes to occur.

Figure S1 Leave-One-Out Forest plot illustrating odds ratio of COVID-19-related outcomes in patients with severe mental illness

(A) COVID-19 infection

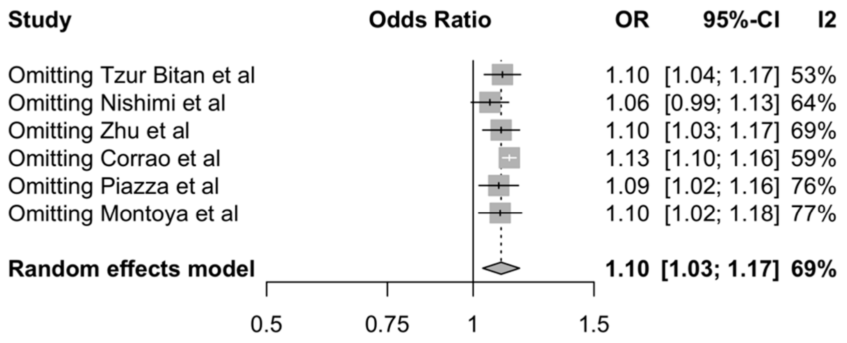

(B) COVID-19-related hopitalization

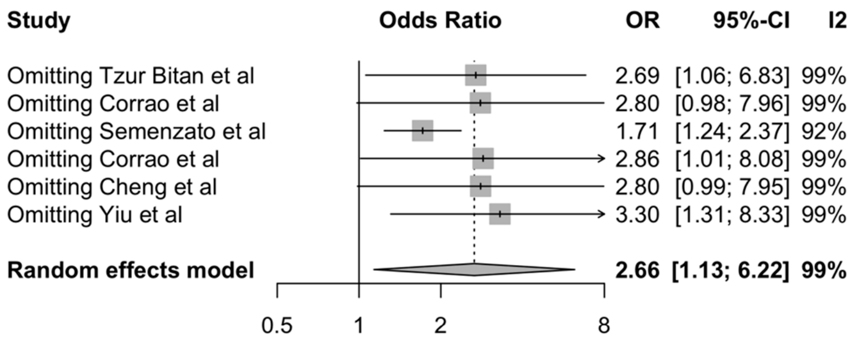

(C) COVID-19-related mortality

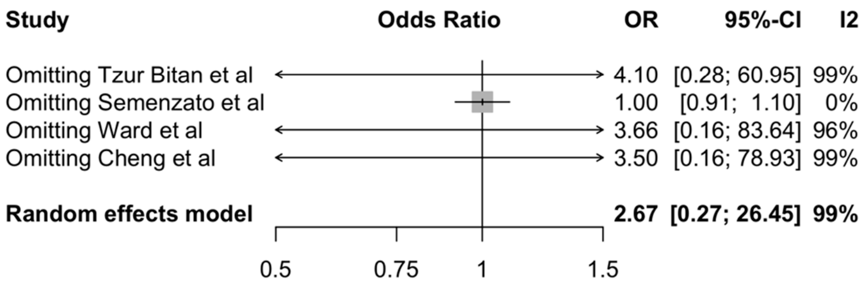

Figure S2 Funnel plot for publication bias for pooled COVID-19-related outcomes post vaccination in patients with severe mental illness

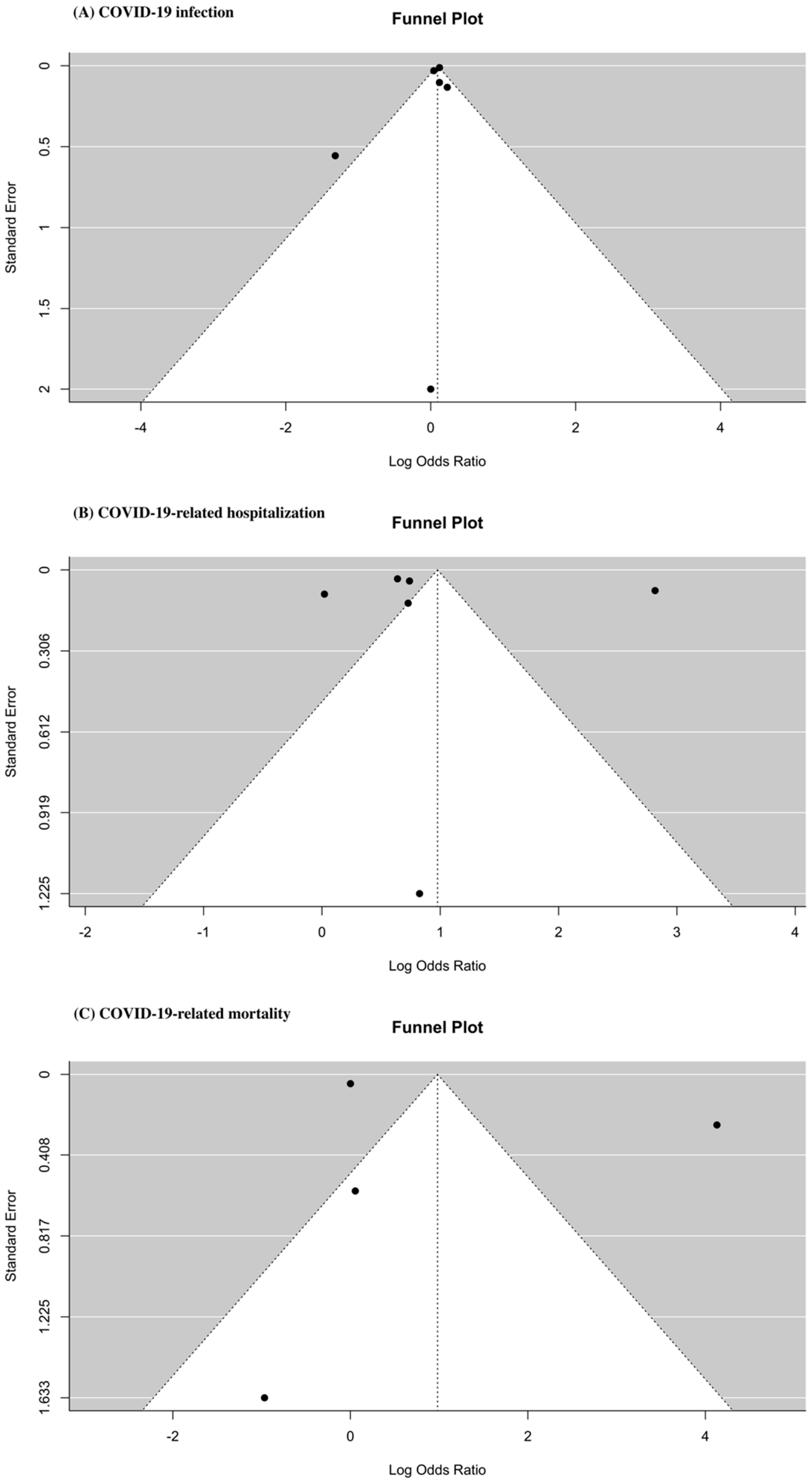

Table S3 Results of Egger test for COVID-19-related outcomes

Outcome	*Z*	P
**COVID-19 Infection**	-1.063	0.29
**COVID-19-related Hospitalization**	-0.097	0.92
**COVID-19-related Mortality**	-0.744	0.46

Reference

1. Tzur Bitan D, Kridin K, Cohen AD, Weinstein O. COVID-19 hospitalisation, mortality, vaccination, and postvaccination trends among people with schizophrenia in Israel: a longitudinal cohort study. *Lancet Psychiatry* **2021**, 8(10), 901-908.
2. Semenzato L, Botton J, Drouin J, Baricault B, Bertrand M, Jabagi M-J, Cuenot F, Vu SL, Dray-Spira R, Weill A *et al.* Characteristics associated with the residual risk of severe COVID-19 after a complete vaccination schedule: A cohort study of 28 million people in France. *Lancet Reg Health Eur* **2022**, 19, 100441.
3. Nishimi K, Neylan TC, Bertenthal D, Seal KH, O'Donovan A. Association of Psychiatric Disorders With Incidence of SARS-CoV-2 Breakthrough Infection Among Vaccinated Adults. *JAMA Netw Open* **2022**, 5(1), e227287.
4. Piazza MF, Amicizia D, Marchini F, Astengo M, Grammatico F, Battaglini A, Sticchi C, Paganino C, Lavieri R, Andreoli GB *et al.* Who Is at Higher Risk of SARS-CoV-2 Reinfection? Results from a Northern Region of Italy. *Vaccines (Basel)* **2022**, 10(11), 1885.
5. Corrao G, Franchi M, Cereda D, Bortolan F, Leoni O, Borriello CR, Della Valle PG, Tirani M, Pavesi G, Barone A *et al.* Vulnerability Predictors of Post-Vaccine SARS-CoV-2 Infection and Disease—Empirical Evidence from a Large Population-Based Italian Platform. *Vaccines (Basel)* **2022**, 10(6), 845.
6. Corrao G, Franchi M, Cereda D, Bortolan F, Leoni O, Jara J, Valenti G, Pavesi G. Factors associated with severe or fatal clinical manifestations of SARS-CoV-2 infection after receiving the third dose of vaccine. *J Intern Med* **2022**, 292(5), 829-836.
7. Zhu J, Tian H, Wang H, Yu H, Liu C, Wang L, Li Q, Fang T, Jia F, Li Y *et al.* Higher benefit-risk ratio of COVID-19 vaccination in patients with schizophrenia and major depressive disorder versus patients with bipolar disorder when compared to controls. *Am J Transl Res* **2022**, 14(8), 5719-5729.
8. Montoya A, Wen K, Travers JL, Rivera-Hernandez M, White E, Mor V, Berry SD. Resident Factors Associated With Breakthrough SARS-CoV-2 Infections. *J Am Med Dir Assoc* **2023**, S1525-8610(23)00225-6.

9. Ward I, Robertson C, Agrawal U, Patterson L, Bradley DT, Shi T, de Lusignan S, Hobbs R, Sheikh A, Nafilyan V. Risk of COVID-19 death in adults who received booster COVID-19 vaccinations in England. *Nature communications* **2024**, 15(1), 398.
10. Cheng WJ, Shih HM, Su KP, Hsueh PR. Risk factors for poor COVID-19 outcomes in patients with psychiatric disorders. *Brain Behav Immun* **2023**, 114, 255-261.
11. Yiu HHE, Yan VKC, Wei Y, Ye X, Huang C, Castle DJ, Chui CSL, Lai FTT, Li X, Wong CKH *et al*. Risks of COVID-19-related hospitalisation and mortality among individuals with mental disorders following BNT162b2 and CoronaVac vaccinations: A case-control study. *Psychiatry research* **2023**, 329, 115515.
